# Effects of Sodium-Glucose Cotransporter-2 Inhibitors on Urine Albumin to Creatinine Ratio in Type 2 Diabetes Mellitus Patients and Medication Care

**DOI:** 10.1155/2022/5854200

**Published:** 2022-07-20

**Authors:** Dong-Dong Wang, Cun Zhang, Yang Yang, Su-Mei He, Ping Zhu, Xiao Chen

**Affiliations:** ^1^Jiangsu Key Laboratory of New Drug Research and Clinical Pharmacy & School of Pharmacy, Xuzhou Medical University, Xuzhou, Jiangsu 221004, China; ^2^Department of Pharmacy, Xuzhou Oriental Hospital Affiliated to Xuzhou Medical University, Xuzhou, Jiangsu 221004, China; ^3^Department of Pharmacy, The Affiliated Changzhou Children's Hospital of Nantong University, Changzhou 213003, China; ^4^Department of Pharmacy, The Affiliated Suzhou Science & Technology Town Hospital of Nanjing Medical University, Suzhou Jiangsu 215153, China; ^5^Department of Endocrinology, Huaian Hospital of Huaian City, Huaian, Jiangsu 223200, China; ^6^School of Nursing, Xuzhou Medical University, Xuzhou, Jiangsu 221004, China

## Abstract

**Objectives:**

The purpose of this study was to explore the effects of sodium-glucose cotransporter-2 (SGLT-2) inhibitors on urine albumin to creatinine ratio (UACR) in type 2 diabetes mellitus (T2DM) patients and to recommend appropriate medication care scheme.

**Methods:**

8371 T2DM patients from four dapagliflozin studies and two canagliflozin studies were collected for analyzing with nonlinear mixed effect model (NONMEM). The change rates of UACR from baseline were intended to be evaluation indicators.

**Results:**

In the present study, there was no significant difference in the effects on UACR using dapagliflozin or canagliflozin treatment in T2DM patients. The maximal effect (*E*_max_) and the treatment duration of reaching half of *E*_max_ (ET_50_) from SGLT-2 inhibitors on UACR in T2DM patients were -19.2% and 0.448 weeks, respectively. Further, the treatment duration to reach 25%, 50%, 75%, and 80% *E*_max_ was 0.150 weeks, 0.448 weeks, 1.344 weeks, and 1.792 weeks, respectively. Namely, for achieving the plateau period (80% of *E*_max_) of SGLT-2 inhibitors on UACR in T2DM patients, 10 mg/day dapagliflozin (or 100 mg/day canagliflozin) should be taken for at least 1.792 weeks.

**Conclusions:**

To our knowledge, the present study explored the effects of SGLT-2 inhibitors on UACR in T2DM patients, meanwhile, recommended appropriate medication care scheme for the first time.

## 1. Introduction

Diabetes mellitus (DM) was a serious disease threatening to human health and a public health problem attracting more and more worldwide attention [1]. It was reported that in 2010, the estimated prevalence of DM in adults worldwide was 6.4%, and in 2030, there would be approximately 7.7% population suffering from DM in the world [2, 3]. China accounted for about 30% in the world, of which type 2 DM (T2DM) accounted for 90% [1]. What was more important was that DM could be complicated with multiple diseases significantly increasing the death risk of DM patient [1]. As everyone knows, long-term hyperglycemia would result in chronic damage and dysfunction of various tissues, especially kidneys [4], blood vessels [5], nerves [6], and heart [7], among which kidney damage was one of the most common microvascular complications in DM patients, which brought great challenges to the treatment and nursing for DM patients.

Urine albumin to creatinine ratio (UACR), also known as urine microalbumin, helps identify kidney diseases that could occur as a complication of DM [8]. At present, more and more studies had used UACR as a valuable evaluation index for kidney damage in T2DM patients [9–11]. Sodium-glucose cotransporter-2 (SGLT-2) inhibitors was a group of antidiabetic drugs and play a hypoglycemic role by restraining SGLT-2 who was accounted for approximately 90% glucose absorption in the kidney [12–14]. In recent years, it had been reported that SGLT-2 inhibitors had favourable renal protective effect and safety [15]. However, the influences from SGLT-2 inhibitors on UACR in T2DM patients remained unknown. The purpose of this study was to explore the effects of SGLT-2 inhibitors on UACR in T2DM patients and to recommend appropriate medication care scheme.

## 2. Methods

### 2.1. Included Patients

**Figure 1:**
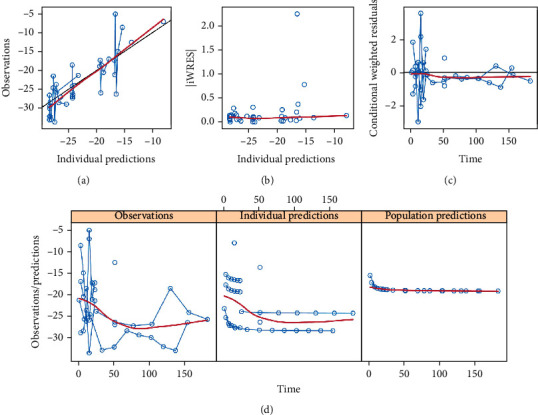
Model evaluation. (a) Observations vs. individual predictions, (b) absolute value of individual weighted residuals (│iWRES│) vs. individual predictions, (c) conditional weighted residuals (CWRES) vs. time, and (d) observations/predictions vs. time.

T2DM patients with treatment using SGLT-2 inhibitors from published literatures were included to analyze [16–21]. Supplementary showed literature search program and detailed inclusion information such as source, group, dosage, duration of treatment, number of people, and age (Table 1S was search details, Figure 1S was search strategies, and Table 2S was identified studies). The change rates of UACR from baseline were intended to be evaluation indicators for eliminating the potential baseline effect, which was shown in the following formula:

(1) $$\begin{array}{c} U=\frac{U_{\text{time}}-U_{\text{base}}}{U_{\text{base}}}\times 100\%. \end{array}$$

*U* was the change rate of UACR from baseline; *U*_time_ was the value of UACR at time; *U*_base_ was the value of UACR at baseline.

### 2.2. Model Establishment

The placebo control group effects were eliminated from the sum effects to obtain the actual SGLT-2 inhibitors effects on UACR in T2DM patients. In addition, *E*_max_ model was used to evaluate the effects of SGLT-2 inhibitors on UACR in T2DM patients, shown in the following formulas:

(2) $$\begin{array}{c} U_{a,i,j}=U_{s,i,j}-U_{p,i,j}, \end{array}$$

(3) $$\begin{array}{c} U_{a,i,j}=\frac{E_{\max,\;i,j}\times \text{Time}}{{\text{ET}}_{50,i,j}+\text{Time}}+\frac{{Ɛ}_{i,j}}{\sqrt{N_{i,j}/1000}}. \end{array}$$

*U*_*s*,*i*,*j*_ was the sum effects of SGLT-2 inhibitors on UACR in T2DM patients; *U*_*p*,*i*,*j*_ was the placebo control group effects on UACR in T2DM patients; *U*_*a*,*i*,*j*_ was the actual effects of SGLT-2 inhibitors on UACR in T2DM patients; *i* was different studies; *j* was time point. *E*_max_ was the maximal effects of SGLT-2 inhibitors on UACR in T2DM patients; ET_50_ was the treatment time to achieve half of the *E*_max_; *Ɛ*_*i*,*j*_ was the residual error; *N*_*i*,*j*_ was the sample size.

Formulas (4)–(7) showed variabilities of interstudy which were described by exponential or additive error models:

(4) $$\begin{array}{c} E_{\max,i,j}=E_{\max}\times \exp\left(b_{1,i}\right), \end{array}$$

(5) $$\begin{array}{c} {\text{ET}}_{50,i,j}={\text{ET}}_{50}\times \exp\left(b_{2,i}\right), \end{array}$$

(6) $$\begin{array}{c} E_{\max,i,j}=E_{\max}+b_{1,i}, \end{array}$$

(7) $$\begin{array}{c} {\text{ET}}_{50,i,j}={\text{ET}}_{50}+b_{2,i}. \end{array}$$

*b*_1,*i*_ and *b*_2,*i*_ were the interstudy variabilities.

Formulas (8)–(10) showed continuous or categorical covariates:

(8) $$\begin{array}{c} U_{i}=U_{T}+\left(\text{COV}-{\text{COV}}_{m}\right)\cdot \theta_{c}, \end{array}$$

(9) $$\begin{array}{c} U_{i}=U_{T}\times {\left(\frac{\text{COV}}{{\text{COV}}_{m}}\right)}^{\theta_{c}}, \end{array}$$

(10) $$\begin{array}{c} U_{i}=U_{T}+\text{COV}\times \theta_{c}. \end{array}$$

*U*_*i*_ was individual parameter; *U*_*T*_ was typical parameter; COV was covariate; COV_*m*_ was median value. *θ*_*c*_ was correction coefficient. Different SGLT-2 inhibitors and dosages were also selected as potential covariables to evaluate whether there were significant difference on UACR in T2DM patients between different drugs or different dosages.

The nonlinear mixed effect modeling (NONMEM) software was used for building up model. Once basic model was done, potential covariate was considered for adding into *E*_max_ or ET_50_. The objective function value (OFV) change was used as covariate inclusion criteria, when OFV decreased more than 3.84 (*χ*^2^, *α* = 0.05, d.f. = 1), it was considered sufficient for inclusion, when OFV increased more than 6.63 (*χ*^2^, *α* = 0.01, d.f. = 1), it was considered sufficient for significance in the final model [22].

### 2.3. Model Evaluation

The observations vs. individual predictions, absolute value of individual weighted residuals (│iWRES│) vs. individual predictions, conditional weighted residuals (CWRES) vs. time, observations/predictions vs. time, individual plots, density vs. CWRES, and quantiles of CWRES vs. quantiles of normal were used to evaluate the final model. The visual predictive check (VPC) plot was used to assess the predictive performance of final model. The Bootstrap was used to assess the stability of model.

### 2.4. Prediction

The curve from the final model of effects of SGLT-2 inhibitors on UACR in T2DM patients was simulated, including the duration time achieving 25%, 50%, 75%, and 80% Emax of SGLT-2 inhibitors on UACR in T2DM.

## 3. Results

### 3.1. Included Patients

8371 T2DM patients from four dapagliflozin studies and two canagliflozin studies were collected for analysis [16–21], including five 10 mg/day dapagliflozin groups, two 100 mg/day canagliflozin groups, and one 300 mg/day canagliflozin group. Detailed information were shown in Supplementary. The vast majority of these studies were multinational sources, and their duration of treatment were from 16 weeks to 182 weeks.

### 3.2. Modeling

The *E*_max_ and ET_50_ from SGLT-2 inhibitors on UACR in T2DM patients were -19.2% and 0.448 weeks, respectively. Furthermore, in terms of different SGLT-2 inhibitors drugs and dosages, 10 mg/day dapagliflozin, 100 mg/day canagliflozin, and 300 mg/day canagliflozin were not covariates included in the final model, indicating there were no significant difference on UACR in T2DM patients from 10 mg/day dapagliflozin, 100 mg/day canagliflozin, or 300 mg/day canagliflozin. In other words, for clinical use, 10 mg/day dapagliflozin or 100 mg/day canagliflozin was available for treatment on UACR in T2DM patients.

The formulas (11) showed the final model of SGLT-2 inhibitors on UACR in T2DM patients:

(11) $$\begin{array}{c} U=\frac{-19.2\%\times \text{Time}}{0.448+\text{Time}}. \end{array}$$

*U* was the change rate of UACR; Time was SGLT-2 inhibitors duration time to treat UACR in T2DM patients.

### 3.3. Evaluation

**Figure 2:**
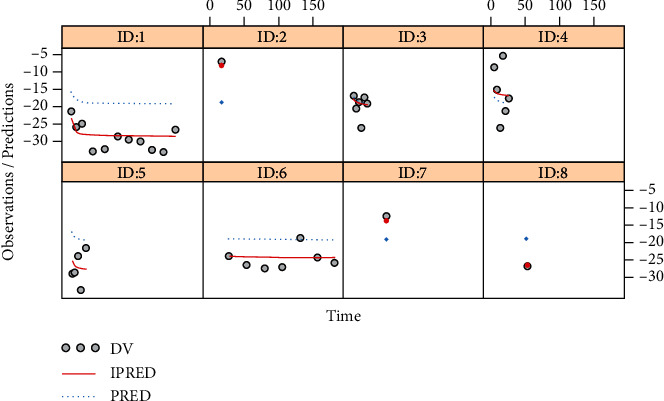
Individual plots. ID: 1-8 were from studies [16–21].

**Figure 3:**
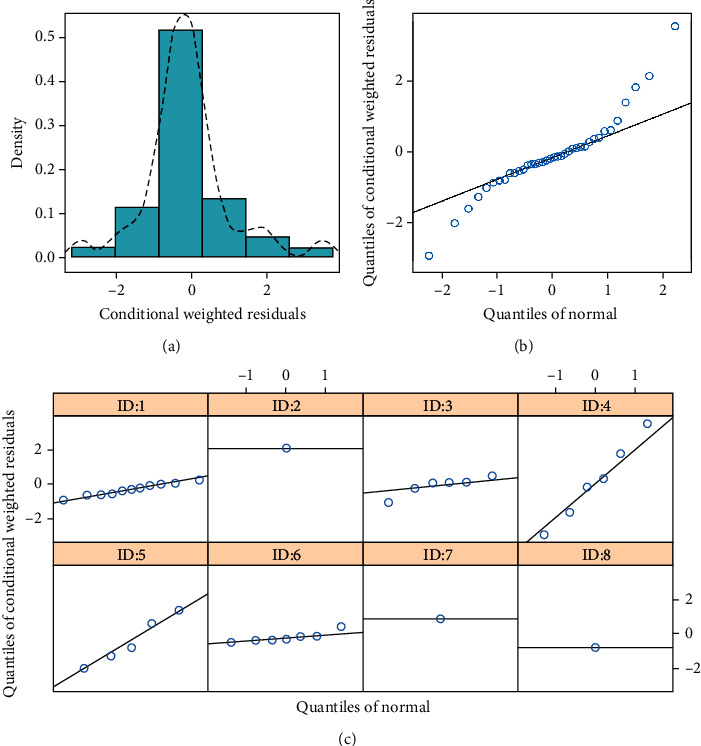
Distribution of conditional weighted residuals for model. (a) Density vs. conditional weighted residuals (CWRES), (b) quantiles of CWRES vs. quantiles of normal, and (c) quantiles of CWRES vs. quantiles of normal for individual. ID: 1-8 were from studies [16–21].

**Figure 4:**
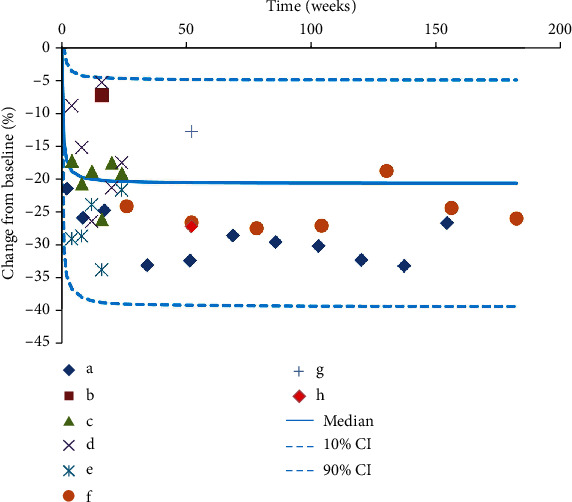
Visual predictive check plots. Median, 10% CI and 90% CI were simulated by Monte Carlo (*n* = 1000); CI: confidence interval. a-h were from studies [16–21].

The final model evaluation was shown in Figures 1, 2, and 3, among which Figure 1 was observations vs. individual predictions,│iWRES│vs. individual predictions, CWRES vs. time, and observations/predictions vs. time; Figure 2 was individual plots; and Figure 3 was density vs. CWRES and quantiles of CWRES vs. quantiles of normal. Overall speaking, individual predictions and observations had better linear relationship. The VPC plot was shown in Figure 4, indicating all observed data were included in the 10-90% prediction intervals produced with simulation data and showing the predictive power of the final model. In addition, the Bootstrap was shown in Table 1, and the absolute values of bias were all less than 30%.

### 3.4. Prediction

**Figure 5:**
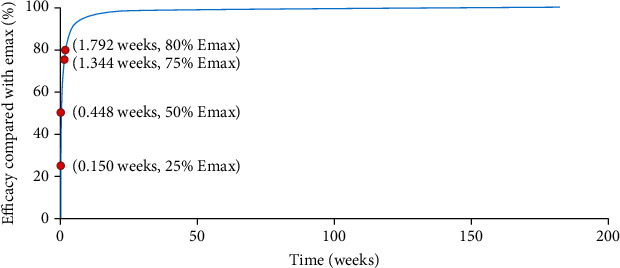
Model prediction.

Figure 5 showed the curve of effects from SGLT-2 inhibitors on UACR in T2DM patients, where the treatment duration to reach 25%, 50%, 75%, and 80% *E*_max_ was 0.150 weeks, 0.448 weeks, 1.344 weeks, and 1.792 weeks, respectively. Namely, for achieving the plateau period (80% of *E*_max_) of SGLT-2 inhibitors on UACR in T2DM patients, 10 mg/day dapagliflozin (or 100 mg/day canagliflozin) should be taken for at least 1.792 weeks.

## 4. Discussion

In the world, the number of DM patients had quadrupled in the past three decades, and meanwhile, DM was the ninth major cause of death, among which Asia had become the major area of the rapidly emerging T2DM global epidemic, and most T2DM patients always had at least one complication [23]. The complexity of T2DM treatment and care were very challenging because they involved the prevention of organ damage and complications [23], including chronic damage and dysfunction of various tissues, especially kidneys [4], blood vessels [5], nerves [6], and heart [7], among which kidney damage was one of the most common microvascular complications in DM patients, which brought great challenges to the treatment and nursing for DM patients.

SGLT-2 inhibitors were a group of antidiabetic drugs, which had the ability to reduce the blood sugar via inhibiting SGLT-2 [24]. Furthermore, except for lowering blood sugar [25–30], SGLT-2 inhibitors also had abilities to lose weight [24, 31, 32], reduce cardiovascular outcomes and mortality risk [33], and play renal protective effect [15]. It was also reported that SGLT-2 inhibitors could observably lower the response of inflammatory and smaller infarct size compared with other oral antidiabetic drugs, not dependent on blood sugar control [34]. In addition, as everyone knows, UACR, also known as urine microalbumin, helps identify kidney disease that could occur as a complication of diabetes [8]. More importantly, numerous studies had used UACR as a valuable evaluation index for kidney damage in T2DM patients [9–11], where the efficacy of treatment could be quantified by analyzing changes in UACR after continuous treatment. However, the effects of SGLT-2 inhibitors on UACR in T2DM patients remained unknown. The purpose of this study was to explore the effects of SGLT-2 inhibitors on UACR in T2DM patients and to recommend appropriate medication care scheme.

In the present study, 8371 T2DM patients from four dapagliflozin studies and two canagliflozin studies were collected for analysis [16–21], including five 10 mg/day dapagliflozin groups, two 100 mg/day canagliflozin groups, and one 300 mg/day canagliflozin group. The change rates of UACR from baseline were intended to be evaluation indicators for eliminating the potential baseline effect. Additionally, the placebo control group effects were eliminated from the sum effects to obtain the actual SGLT-2 inhibitors effects on UACR in T2DM patients, and *E*_max_ model was used to evaluate the effects of SGLT-2 inhibitors on UACR in T2DM patients.

Through model analysis, this study finally found that the *E*_max_ and ET_50_ from SGLT-2 inhibitors on UACR in T2DM patients were -19.2% and 0.448 weeks, respectively. Furthermore, in terms of different SGLT-2 inhibitors drugs and dosages, 10 mg/day dapagliflozin, 100 mg/day canagliflozin, and 300 mg/day canagliflozin were not covariates included in the final model, indicating there were no significant difference on UACR in T2DM patients from 10 mg/day dapagliflozin, 100 mg/day canagliflozin, or 300 mg/day canagliflozin. In addition, the lack of a dose-response relationship between SGLT2 inhibitors and a series of safety or efficacy outcomes had been already indicated in Mirabelli et al.'s study [35]. In other words, for clinical use, 10 mg/day dapagliflozin or 100 mg/day canagliflozin was available for treatment on UACR in T2DM patients. Additionally, the present study simulated the curve from the final model of effects of SGLT-2 inhibitors on UACR in T2DM patients including the duration time achieving 25%, 50%, 75%, and 80% *E*_max_ of SGLT-2 inhibitors on UACR in T2DM and found that the treatment duration to reach 25%, 50%, 75%, and 80% *E*_max_ was 0.150 weeks, 0.448 weeks, 1.344 weeks, and 1.792 weeks, respectively. That was to say, for achieving the plateau period (80% of *E*_max_) of SGLT-2 inhibitors on UACR in T2DM patients, 10 mg/day dapagliflozin (or 100 mg/day canagliflozin) should be taken for at least 1.792 weeks, which could provide reference for clinical medication care.

However, there were also objective limitations in the present study. As the number of relevant studies about SGLT-2 inhibitors on UACR in T2DM patients were limited on account of the current SGLT-2 inhibitors treatment for UACR in T2DM patients was a new discovery. In addition, most original studies that had looked at the effects of SGLT2 inhibitors on UACR had relied on post hoc analysis, and its calculations of ET_50_ and *E*_max_ may require farther confirmation and validation in future investigations.

## 5. Conclusion

To our knowledge, the present study explored the effects of SGLT-2 inhibitors on UACR in T2DM patients, meanwhile, recommended appropriate medication care scheme for the first time.

## Figures and Tables

**Table 1:** Parameter estimates of final model and Bootstrap.

Parameter	Estimate	Bootstrap (*n* = 1000)		Bias (%)
		Median	(Lower quartile, upper quartile)	
*E*_max_, %	-19.2	-22.6	(-39.6, -18.6)	17.71
ET_50_, week	0.448	0.575	(0.010, 1.530)	28.35
*ω*_*E*max_	0.423	0.453	(0.255, 0.642)	7.09
*Ɛ*	9.965	9.750	(6.033, 12.845)	-2.16

*E*_max_ was the maximal effect; ET_50_ was the treatment duration to reach half of *E*_max_; *ω*_*E*max_ was the interstudy variability of *E*_max_; *Ɛ* was the residual error; Bias = (Median − Estimate)/Estimate × 100%.

## Data Availability

The data related to this article can be publicly available after the article accepted.

## Supplementary Materials

Supplementary Materials The Supplementary Materials have been submitted along with the primary manuscript, including search strategy and studies identified for analysis.
