# Supplementary material for: Effects of Sodium-Glucose Cotransporter-2 Inhibitors on Urine Albumin to Creatinine Ratio in Type 2 Diabetes Mellitus Patients and Medication Care
Source: J Diabetes Res. 2022 Jul 20;2022:5854200. doi: 10.1155/2022/5854200 (PMC9328955; doi:10.1155/2022/5854200)
Supplement: Supplementary Materials — The Supplementary Materials have been submitted along with the primary manuscript, including search strategy and studies identified for analysis. [file 5854200.f1.docx]

**Table 1S. Search details**

| No. | Query | Results |
| --- | --- | --- |
| #1 | canagliflozin[Title] | 631 |
| #2 | dapagliflozin[Title] | 1,013 |
| #3 | empagliflozin[Title] | 944 |
| #4 | ertugliflozin[Title] | 98 |
| #5 | ipragliflozin[Title] | 172 |
| #6 | luseogliflozin[Title] | 71 |
| #7 | tofogliflozin[Title] | 89 |
| #8 | type 2 diabetes[Title] | 67,665 |
| #9 | type 2 diabetes mellitus[Title] | 20,457 |
| #10 | T2D[Title] | 187 |
| #11 | T2DM[Title] | 833 |
| #12 | type 2 diabetic patients[Title] | 4,368 |
| #13 | urine albumin to creatinine ratio | 4,545 |
| #14 | UACR | 1400 |
| #15 | #1 OR #2 OR #3 OR #4 OR #5 OR #6 OR #7 | 2,970 |
| #16 | #8 OR #9 OR #10 OR #11 OR #12 | 72,836 |
| #17 | #13 OR #14 | 5,074 |
| #18 | #15 AND #16 AND #17 | 28 |
| #19 | #18 Filters: Humans | 21 |

**Figure 1S. Strategy for literature search**

**List of final included studies (n=6)**

**Dapagliflozin (n=4)**

1. Jongs N, Greene T, Chertow GM, McMurray JJV, Langkilde AM, Correa-Rotter R, Rossing P, Sjöström CD, Stefansson BV, Toto RD, Wheeler DC, Heerspink HJL; DAPA-CKD Trial Committees and Investigators. Effect of dapagliflozin on urinary albumin excretion in patients with chronic kidney disease with and without type 2 diabetes: a prespecified analysis from the DAPA-CKD trial. Lancet Diabetes Endocrinol. 2021 Nov;9(11):755-766. doi: 10.1016/S2213-8587(21)00243-6. Epub 2021 Oct 4. PMID: 34619106.

2. van Ruiten CC, van der Aart-van der Beek AB, IJzerman RG, Nieuwdorp M, Hoogenberg K, van Raalte DH, Heerspink HJL. Effect of exenatide twice daily and dapagliflozin, alone and in combination, on markers of kidney function in obese patients with type 2 diabetes: A prespecified secondary analysis of a randomized controlled clinical trial. Diabetes Obes Metab. 2021 Aug;23(8):1851-1858. doi: 10.1111/dom.14410. Epub 2021 May 14. PMID: 33908691; PMCID: PMC8360098.

3. Scholtes RA, van Raalte DH, Correa-Rotter R, Toto RD, Heerspink HJL, Cain V, Sjöström CD, Sartipy P, Stefánsson BV. The effects of dapagliflozin on cardio-renal risk factors in patients with type 2 diabetes with or without renin-angiotensin system inhibitor treatment: a post hoc analysis. Diabetes Obes Metab. 2020 Apr;22(4):549-556. doi: 10.1111/dom.13923. Epub 2019 Dec 14. PMID: 31742881; PMCID: PMC7078964.

4. Pollock C, Stefánsson B, Reyner D, Rossing P, Sjöström CD, Wheeler DC, Langkilde AM, Heerspink HJL. Albuminuria-lowering effect of dapagliflozin alone and in combination with saxagliptin and effect of dapagliflozin and saxagliptin on glycaemic control in patients with type 2 diabetes and chronic kidney disease (DELIGHT): a randomised, double-blind, placebo-controlled trial. Lancet Diabetes Endocrinol. 2019 Jun;7(6):429-441. doi: 10.1016/S2213-8587(19)30086-5. Epub 2019 Apr 13. PMID: 30992195.

**Canagliflozin (n=2)**

5. Wada T, Mori-Anai K, Kawaguchi Y, Katsumata H, Tsuda H, Iida M, Arakawa K, Jardine MJ. Renal, cardiovascular and safety outcomes of canagliflozin in patients with type 2 diabetes and nephropathy in East and South-East Asian countries: Results from the Canagliflozin and Renal Events in Diabetes with Established Nephropathy Clinical Evaluation Trial. J Diabetes Investig. 2022 Jan;13(1):54-64. doi: 10.1111/jdi.13624. Epub 2021 Aug 3. PMID: 34212533; PMCID: PMC8756319.

6. Yale JF, Bakris G, Cariou B, Nieto J, David-Neto E, Yue D, Wajs E, Figueroa K, Jiang J, Law G, Usiskin K, Meininger G; DIA3004 Study Group. Efficacy and safety of canagliflozin over 52 weeks in patients with type 2 diabetes mellitus and chronic kidney disease. Diabetes Obes Metab. 2014 Oct;16(10):1016-27. doi: 10.1111/dom.12348. Epub 2014 Jul 22. PMID: 24965700.

**Table 2S. Studies identified for analysis**

| Studies | Sources | Groups | Dosage | Duration of treatment | Number of people | Age  (years) |
| --- | --- | --- | --- | --- | --- | --- |
| Jongs (2021) | Multinational | Dapagliflozin | 10 mg/day | 36 months | 1455 | 60.9-62.7 |
|  |  | Placebo | -- | 36 months | 1451 | 60.9-62.7 |
| van Ruiten (2021) | Netherlands | Dapagliflozin | 10 mg/day | 16 weeks | 16 | 64.1 (8.4) |
|  |  | Placebo | -- | 16 weeks | 17 | 61.5 (7.2) |
| Scholtes (2020) | Multinational | Dapagliflozin 1 | 10 mg/day | 24 weeks | 470 | 61.1 (9.2) |
|  |  | Placebo 1 | -- | 24 weeks | 481 | 61.6 (8.4) |
|  |  | Dapagliflozin 2 | 10 mg/day | 24 weeks | 148 | 55.4 (10.8) |
|  |  | Placebo 2 | -- | 24 weeks | 146 | 56.8 (11.2) |
| Pollock (2019) | Multinational | Dapagliflozin | 10 mg/day | 24 weeks | 141 | 64.7 (8.6) |
|  |  | Placebo | -- | 24 weeks | 145 | 64.7 (8.5) |
| Wada (2022) | Multinational | Canagliflozin | 100 mg/day | 182 weeks | 1817 | 60.8-63.4 |
|  |  | Placebo | -- | 182 weeks | 1815 | 60.8-63.4 |
| Yale (2014) | Multinational | Canagliflozin 1 | 100 mg/day | 52 weeks | 90 | 69.5 (8.2) |
|  |  | Canagliflozin 2 | 300 mg/day | 52 weeks | 89 | 67.9 (8.2) |
|  |  | Placebo | -- | 52 weeks | 90 | 68.2 (8.4) |
